# The Effect of Antitumor Glycosides on Glioma Cells and Tissues as Studied by Proton HR-MAS NMR Spectroscopy

**DOI:** 10.1371/journal.pone.0078391

**Published:** 2013-10-23

**Authors:** Isabel García-Álvarez, Leoncio Garrido, Lorenzo Romero-Ramírez, Manuel Nieto-Sampedro, Alfonso Fernández-Mayoralas, Ramón Campos-Olivas

**Affiliations:** 1 Unidad de Neurología Experimental, Hospital Nacional de Parapléjicos, Servicio de Salud de Castilla-La Mancha (SESCAM), Toledo, Spain; 2 Instituto de Química Orgánica General, Consejo Superior de Investigaciones Científicas (CSIC), Madrid, Spain; 3 Instituto de Ciencia y Tecnología de Polímeros, Consejo Superior de Investigaciones Científicas (CSIC), Madrid, Spain; 4 Instituto Cajal de Neurobiología, Consejo Superior de Investigaciones Científicas (CSIC), Madrid, Spain; 5 Spectroscopy and NMR Unit, Structural Biology and Biocomputing Programme, Spanish National Cancer Center (CNIO), Madrid, Spain; National Research Council of Italy, Italy

## Abstract

The effect of the treatment with glycolipid derivatives on the metabolic profile of intact glioma cells and tumor tissues, investigated using proton high resolution magic angle spinning (^1^H HR-MAS) nuclear magnetic resonance (NMR) spectroscopy, is reported here. Two compounds were used, a glycoside and its thioglycoside analogue, both showing anti-proliferative activity on glioma C6 cell cultures; however, only the thioglycoside exhibited antitumor activity *in vivo*. At the drug concentrations showing anti-proliferative activity in cell culture (20 and 40 µM), significant increases in choline containing metabolites were observed in the ^1^H NMR spectra of the same intact cells. *In vivo* experiments in nude mice bearing tumors derived from implanted C6 glioma cells, showed that reduction of tumor volume was associated with significant changes in the metabolic profile of the same intact tumor tissues; and were similar to those observed in cell culture. Specifically, the activity of the compounds is mainly associated with an increase in choline and phosphocholine, in both the cell cultures and tumoral tissues. Taurine, a metabolite that has been considered a biomarker of apoptosis, correlated with the reduction of tumor volume. Thus, the results indicate that the mode of action of the glycoside involves, at least in part, alteration of phospholipid metabolism, resulting in cell death.

## Introduction

The strategy of applying analytical chemistry methods to monitor anti-cancer therapy effects is based on the principle that the interaction of drugs with cells and tissues affects the network of metabolic pathways occurring within cells. This interaction could change the concentration of metabolites associated with the altered pathway and proton high resolution (^1^H HR) NMR spectroscopy offers the possibility of acquiring a snapshot of the sample chemistry, providing, in a single measurement, qualitative and quantitative information of hundreds of metabolites. The complete spectrum can be used as a fingerprint of the metabolic status of cells or tissues [1]. 

Analysis of intact tissue using ^1^H HR magic angle spinning (MAS) NMR spectroscopy provides spectra with high resolution and requires minimal sample preparation, allowing the observation of tissue metabolites in their native state [2,3]. These advantages, together with the potential of ^1^H HR-MAS NMR spectroscopy to identify biomarkers for cancer diagnosis, prognosis and evaluation of therapies, have lead to an increasing use of this methodology during the last decade [4]. Complementary with that, *in vitro* NMR spectroscopy studies of cancer cells, intact and after drug treatment, allow the systematic determination of a large number of metabolites which may provide valuable information on cellular processes. The comparison of the results of NMR spectroscopy studies of cell cultures and tumor biopsies thus facilitates the correlative interpretation of *in vitro* and *in vivo* drug activity, and allows evaluation of the suitability and limitations of each model system.

**Figure 1:**
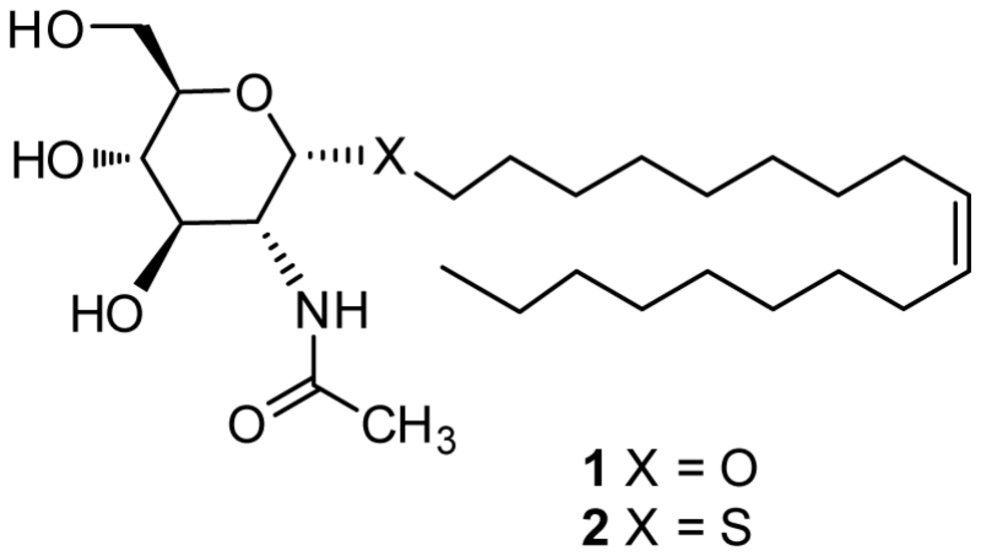
Chemical structures of glycoside 1 and thioglycoside 2.

We have previously described the synthesis of glycolipids with antitumor activity [5]. Among the variety of glycosides with different hydrocarbon chains in the aglycone moiety, the most active glycoside was compound **1**, which contains an oleyl chain (Figure 1). The favorable effect of an oleyl chain on the antitumor activity was also observed in a family of alkylated iminosugars [6]. Further studies [7,8] designed to understand the mechanism behind the activity of **1** on lung carcinoma A549 and glioma C6 cells showed that, at concentrations above 30 µM, glycoside **1** drastically altered the cell lipid profile, with a significant increase in sphingolipid and glycosphingolipid levels, that eventually resulted in cell death [8]. Despite its promising activity against cancer cells in culture, glycoside **1** was inactive when tested in mice bearing an implanted C6 glioma. This lack of activity was attributed to the hydrolysis of **1** by hexosaminidase enzymes generated by activated macrophages [9]. This hypothesis was confirmed by using the enzyme resistant thioglycoside **2** (Figure 1), which reduced significantly the mice tumor volume compared to controls [9].

The antitumor activity of thioglycoside **2**, together with previous results showing alterations on glycosphingolipid levels in tumor cells after compound **2** treatment, indicate the interest of obtaining more information about its mode of action. Accordingly, we hypothesized that drug induced alteration of lipid content should be reflected in observable changes in related metabolic pathways. The information on quantitative response markers could be of great value for the design and evaluation of new compounds with improved therapeutic properties. With this objective, we have studied changes in low molecular weight metabolites after treatment of C6 glioma cells with 1 and 2, as well as the corresponding mice tumor tissues derived from implanting these cells, using ^1^H HR-MAS NMR.

Therefore, the aim of this study was to determine and compare the metabolic profiles of glycoside-treated and untreated glioma cells and tissues, as reflected in the NMR spectra, to gain information on the metabolic impact of the compounds and to provide a better interpretation for their *in vitro* anti-proliferative and *in vivo* antitumoral activity.

## Materials and Methods

### Ethics Statement

Mice were maintained in the animal house of the Cajal Institute (Madrid, Spain) with *ad libitum* food and water in a 12-hours light/dark cycle. Animals were handled complying with the European Union guidelines for care and handling of experimental animals (86/609/EEC) and the protocols approved by the Cajal Institute animal welfare committee.

### Chemicals

Compounds **1** and **2** were prepared following procedures previously described [7,9]. 

### Cell cultures

Rat glioma cells (C6 line, obtained from European Collection of Cell Cultures, Salisbury, UK) were maintained in DMEM medium (Sigma-Aldrich, St Louis, MO), supplemented with fetal bovine serum (FBS, 10%; Gibco, Paisley, UK), penicillin (50 IU/mL) and streptomycin (50 mg/mL), at 37 °C in a 5% CO_2_ humidified atmosphere. Exponentially growing C6 cells were seeded on six-well plates (Beckton Dickinson, Le Pont de Claix, France), in complete DMEM medium, at a density of 6 x 10^5^ cells/well. The cells were allowed to attach with 5% of CO_2_ at 37 °C overnight, and then the medium was replaced with 2 mL of fresh medium. For treatments, stock solutions of compounds **1**-**2** (100 mM) in EtOH were dissolved in DMEM medium containing EGF (10 ng/mL). Cells were treated with 20 µM or 40 µM of compounds (1,2) for 48 h. After the treatment, cells (4 x 10^5^ - 1.2 x 10^6^ cells/well) were trypsinized, centrifuged at 100 x g for 5 minutes and resuspended in phosphate buffer solution (PBS) at 4 °C for analysis. Each experiment was performed in triplicate, and vehicle-treated cells were used as controls.

### C6 glioma tumor samples

The activity of compounds **1** and **2** on tumor growth was evaluated as previously described [9] using an orthotopic model in female nude mice (Foxn1^nu/nu^, 6-8 weeks old, Harlan Iberica, Barcelona, Spain). Mice were maintained in the animal house of the Cajal Institute with ad libitum food and water in a 12-hours light/dark cycle. Animals were handled complying with the European Union guidelines for care and handling of experimental animals (86/609/EEC) and the protocols approved by the Cajal Institute animal welfare committee. Mice were injected into a flank, subcutaneously, a suspension of 2 x 10^6^ cells of rat glioma C6, grown as indicated in the previous section. Tumor volume was calculated according to the following formula: V (cm^3^) = (L x W^2^ x π)/6, where L is the length, W is the width of the tumor, respectively. When tumors reached 250 mm^3^, the animals were treated with a daily intratumoral injection of the compounds, for 14 days. Stock solutions of the compounds (100 mM) in DMSO were dissolved in PBS containing fatty acid-free BSA (5 mg/mL) for the treatments. Control animals received injections with only the vehicle solution (PBS buffer with BSA and DMSO). Four different groups of mice were studied (control, treated with compound **1** at 10 mg/Kg/day and treated with compound **2** at two doses, 1 and 10 mg/Kg/day). For each group six tumor biopsies corresponding to 6 different animals were obtained (N=6). The animals were sacrificed after 14-days treatments, and the tumors were immediately resected in ice. The tumors were cut in two pieces with a sterile surgical blade under a SMA4 dissecting microscope (Askania). For each tumor, two types of samples were obtained and analyzed separately: one from the core of the tumor (representing approximately 5%-10% of total tumor mass) and another from the periphery. The tumor samples were washed once with deuterated phosphate buffered solution, frozen in liquid nitrogen and stored at -80 °C until use.

### ^1^H HR-MAS NMR spectroscopy of cell cultures and *ex vivo* tumor tissues

^1^H HR-MAS NMR measurements were performed on a Bruker spectrometer operating at 16.4 T (proton Larmor frequency of 700.13 MHz) using a Bruker HR-MAS triple resonance probe. The spectra were acquired at 4 °C to minimize the effect of temperature on tissue stability during the acquisition time and to minimize glycerophosphocholine (GPC) and phosphocholine (PC) conversion to choline (Cho) and reduce variations in some amino acids [10]. Sample spinning at the magic angle was applied at a speed of 6 kHz. The acquisition sequence and parameters were selected to reduce the signals of large and low mobility molecules, essentially T_2_-filtered (CPMG) 1D experiments were recorded with relaxation times of 30 and 300 ms (and echo times of 1.5 and 3 ms) for cells and tissue samples, respectively. Low power presaturation during the interscan delay of 3 s was applied for water suppression. Typically, 64 scans were accumulated using a spectral width of 20 ppm with acquisition times of 2 s (cells) or 1.6 s (tissues), which resulted in 6 min of acquisition time per spectrum. The spectra were processed using MestReNova version 7.0 software (Mestrelab Research, Santiago de Compostela, Spain). All free induction decays were processed with exponential multiplication (0.5 Hz line-broadening) before Fourier transformation, followed by baseline correction. The chemical shifts were referenced as follows: a small amount (5 µL) of 10 mM DSS in D_2_O was added to one sample of intact cells and to one sample of tissue, and referenced to DSS = 0 ppm; then all spectra were processed identically and aligned (using the creatine (Cre) methyl resonance at 3.026 ppm) with respect to the sample with added DSS. Out of the various methods to estimate or measure the intensity of NMR signals [4], we compared peak intensity ratios using the methyl signal of creatine as internal reference. Creatine is commonly used as an internal concentration reference for *in vivo* ^1^H NMR, because its concentration correlates with the number of metabolically active cells and is used as a measure of viable cell number [11].

Cultured cells were resuspended, washed three times with deuterated phosphate buffered solution and centrifuged to form pellets. Typically, for each sample approximately 60 μL of living cell pellets were placed into a 4 mm Ø zirconia rotor. Tumor tissue samples for NMR analysis were thawed in ice, cut in a few small pieces that were placed and compacted into a 4 mm Ø zirconia rotor. For both cell and tissue samples a small amount (5 µL) of D_2_O-phosphate buffer was added for field-frequency lock.

### Statistical analysis

Statistically significant differences [12-14] were evaluated as follows: the normal distribution of values was assessed with the Kolmogorov-Smirnov or Shapiro-Wilk tests and the variance homogeneity with the Levene´s test. Values with a normal distribution and homogeneous variance were compared with unpaired two-tailed Student´s t test or, if more than two comparisons were carried out, with the ANOVA test. For values with normal distribution but non-homogeneous variance, the ANOVA test was carried out not assuming equal variances. Values with non-normal distribution were evaluated with the non-parametric Mann-Whitney´s U test or, if more than two comparisons were carried out, with the Kruskal-Wallis test. Significance level was set to 0.05, and all analyses were carried out with SPSS version 19.0 (SPSS Inc., Chicago, IL, USA). The NMR spectra used to quantify metabolite differences, and those presented to illustrate them, are the most representative of three or six independent experiments for cell culture and tumor tissues, respectively. All data are expressed as average value ± standard deviation (SD).

## Results and Discussion

### NMR analysis of C6 cell cultures

**Figure 2:**
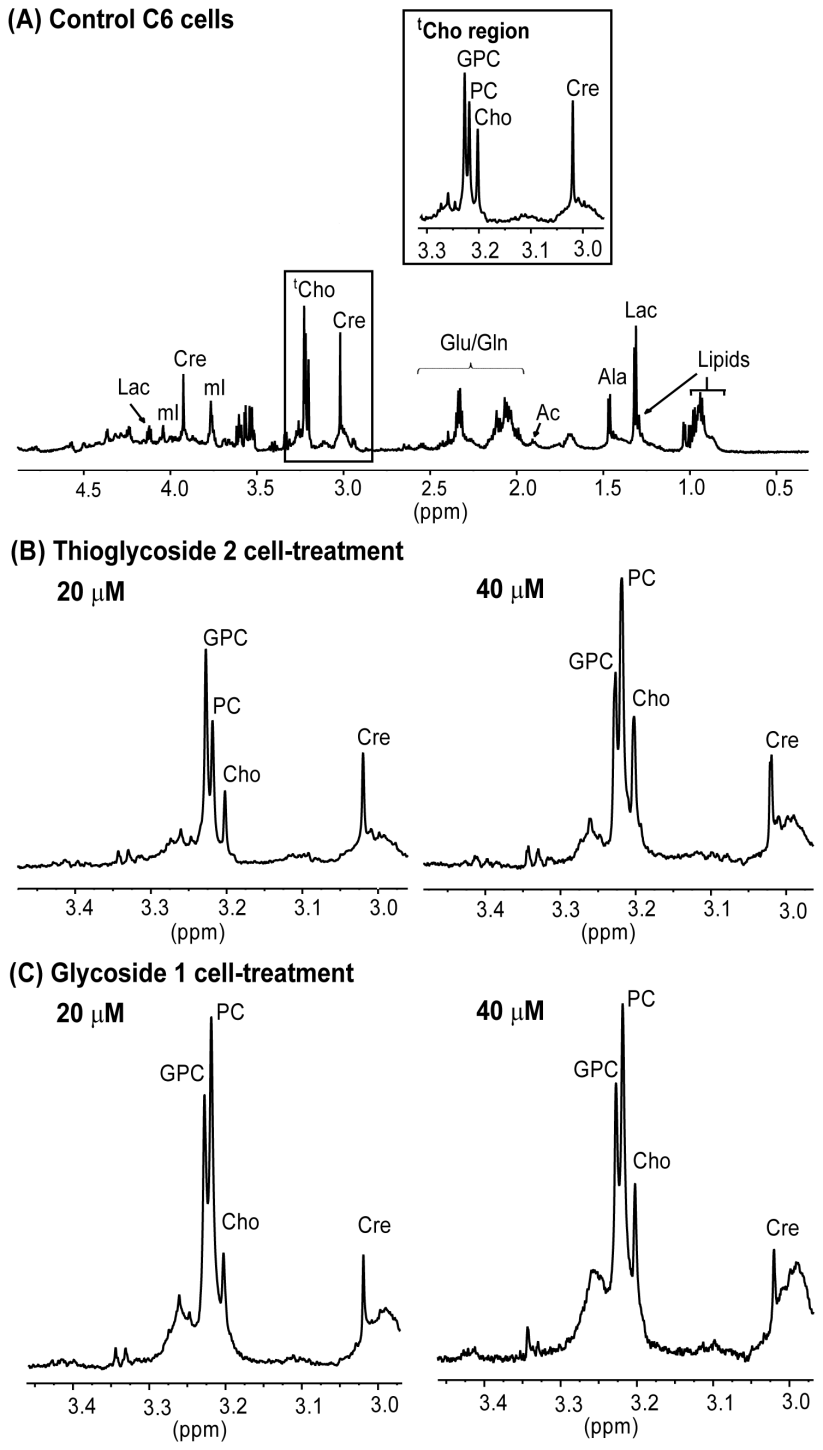
Representative ^1^H HR-MAS NMR spectra of C6 cells. (A) Control C6 cells, with an insert corresponding to the total choline (^t^Cho) region shown on the top (B) ^t^Cho region of the spectrum of C6 cells treated with thioglycoside **2** at 20 and 40 µM (C) ^t^Cho region of the spectrum of C6 cells treated with glycoside **1** at 20 and 40 µM.

The C6 glioma cells were cultured in the presence of two different concentrations (20 and 40 µM) of glycoside **1** or thioglycoside **2** for 48 h. A representative ^1^H NMR spectrum of control C6 glioma cells after 48 h of incubation is shown in Figure 2. Spectra in the range between 0.5 to 4.5 ppm are shown, and the most important variations upon treatment were observed in the region between 3.00 to 3.45 ppm. Low field signals were too weak to allow reliable quantification and were excluded from analysis.

The assignment of relevant, intense and resolved signals was based on the chemical shifts and known multiplicities reported for metabolites of cultured glioma cell lines [11,15], and is illustrated and indicated in a representative spectrum (Figure 2A). Thus, the quartet at 4.12 ppm corresponds to the proton in alpha to the carbonyl group of lactate (Lac). This signal, which was resolved properly, was used to evaluate changes in this metabolite. Lactate presents another ^1^H NMR signal corresponding to its methyl group that appears as a doublet at 1.3 ppm, overlapping with the resonances of lipid methylene groups. The three peaks present in the 3.23 ppm region were assigned to the total choline (^t^Cho) trimethyl resonance, –N(CH_3_)_3_, and consists of at least three main choline subspecies which were differentiated in the HR spectra: choline, phosphocholine and glycerophophocholine at 3.206, 3.223 and 3.231 ppm respectively (Figure 2). Phosphatidylcholine (PdtCho) is a constituent of cell membranes, and this metabolite can be detected by NMR in the region of ^t^Cho at 3.220 ppm and distinguished from other choline-containing metabolites. However, the PdtCho resonance is attenuated to a greater extent than low molecular weight metabolites when using a T_2_-filter, as we did in this study. This sequence also selectively attenuates resonances from macromolecules [16,17]. A narrow peak at 3.02 ppm was assigned to the creatine methyl and employed as internal reference. Signals between 2.34 and 2.04 ppm were assigned to glutamate (Glu) and glutamine (Gln) beta and gamma methylene groups. The singlet at 1.91 ppm was assigned to free acetate (Ac), since the acetate signal corresponding to acetylated species, like *N*-acetyl glucosamine, appears at 2 ppm [18]. A doublet at 1.48 ppm is assigned to alanine (Ala) and signals at 0.90 ppm correspond to methyl groups of fatty acyl chains of lipids (Lip).

While cell-treatment with compounds **1** and **2** produced no significant differences in the levels of aminoacids Glu, Gln, Ala and valine (Val) at any concentration (neither 20 µM nor 40 µM), the levels of other cell metabolites were affected by the treatment, as follows.

Cells treated with the lowest concentration of **2** (20 µM) displayed only slight changes in metabolic profile when compared to control cells. In contrast, the most significant metabolic change, a two-fold increase in ^t^Cho levels compared to creatine, was detected after cell treatment with 40 µM of either glycoside **1** or thioglycoside **2**. Specifically, the levels of Cho and PC increased 2 and 3 times, respectively, and there was a significant rise in the ratio PC/GPC (1.45±0.61 versus 0.70±0.33), due to a greater increase of PC in treated cells (Figure 2, Table 1). Moreover, most of these effects observed in cells treated with glycoside **1** or thioglycoside **2** were concentration-dependent, increasing from 20 to 40 µM level. Previous studies on lipophilic cell extracts had shown that compound **1**, at concentrations above 30 µM, causes increases in ceramide levels and activates endoplasmic reticulum stress response pathways [8], which can induce apoptotic response in cells [17,19]. The reason for this increase of ceramide levels still remains unknown; however, the results obtained in this work are consistent with a buildup of free ceramide due to the hydrolysis of sphingomyelin (SM) catalized by sphingomyelinases [20], contributing to the high levels of PC detected here by NMR spectroscopy upon treatment with compounds **1** and **2**. SM is a major constituent of plasma membranes, and it has been reported that inhibitors of sphingomyelinases block apoptotic cell death in culture [21]. Accordingly, the mode of action of the glycosides used here could involve the direct or indirect functional activation of these sphingomyelinases, resulting in the accumulation of ceramide, which leads to cell apoptosis [22,23]. On the other hand, apoptosis induced by various drugs has been associated with changes in PC in hamster and human cell lines [24], therefore the choline metabolite perturbation detected here could be the final consequences of the treatment (stress response and apoptosis) and not necessarily part of the compounds action pathway.

**Table 1:** Quantification of intracellular metabolites in C6 glioma cells upon glycoside treatment^(a)^.

**Metabolite**	**Signal**	**Control**	**2** (20 µM)	**2** (40 µM)	**1** (20 µM)	**1** (40 µM)
GPC	3.23, s	1.52±0.30	1.77±0.19	1.90±0.43	1.73±0.62	2.42±0.28
PC	3.22, s	1.07±0.21	1.66±0.31	2.76±0.77	2.41±0.72	2.82±0.15
Cho	3.20, s	0.66±0.32	0.76±0.12	1.21±0.43	1.11±0.31	1.87±0.55
^t^Cho	3.2, m	3.25±0.83	4.19±0.62	5.87±1.63	5.25±1.65	7.11±0.98
PC/GPC		0.70±0.33	0.94±0.33	1.45±0.61	1.39±0.80	1.17±0.19
Lac	4.11, q	0.32±0.09	0.19±0.10	0.26±0.10	0.23±0.15	0.32±0.07
Ac	1.91, s	0.16±0.05	0.21±0.10	0.29±0.09	0.31±0.04	0.58±0.13

a Relative intensity to creatine (I/I_creatine_). Results represent the mean ± SD of three experiments.

**Figure 3:**
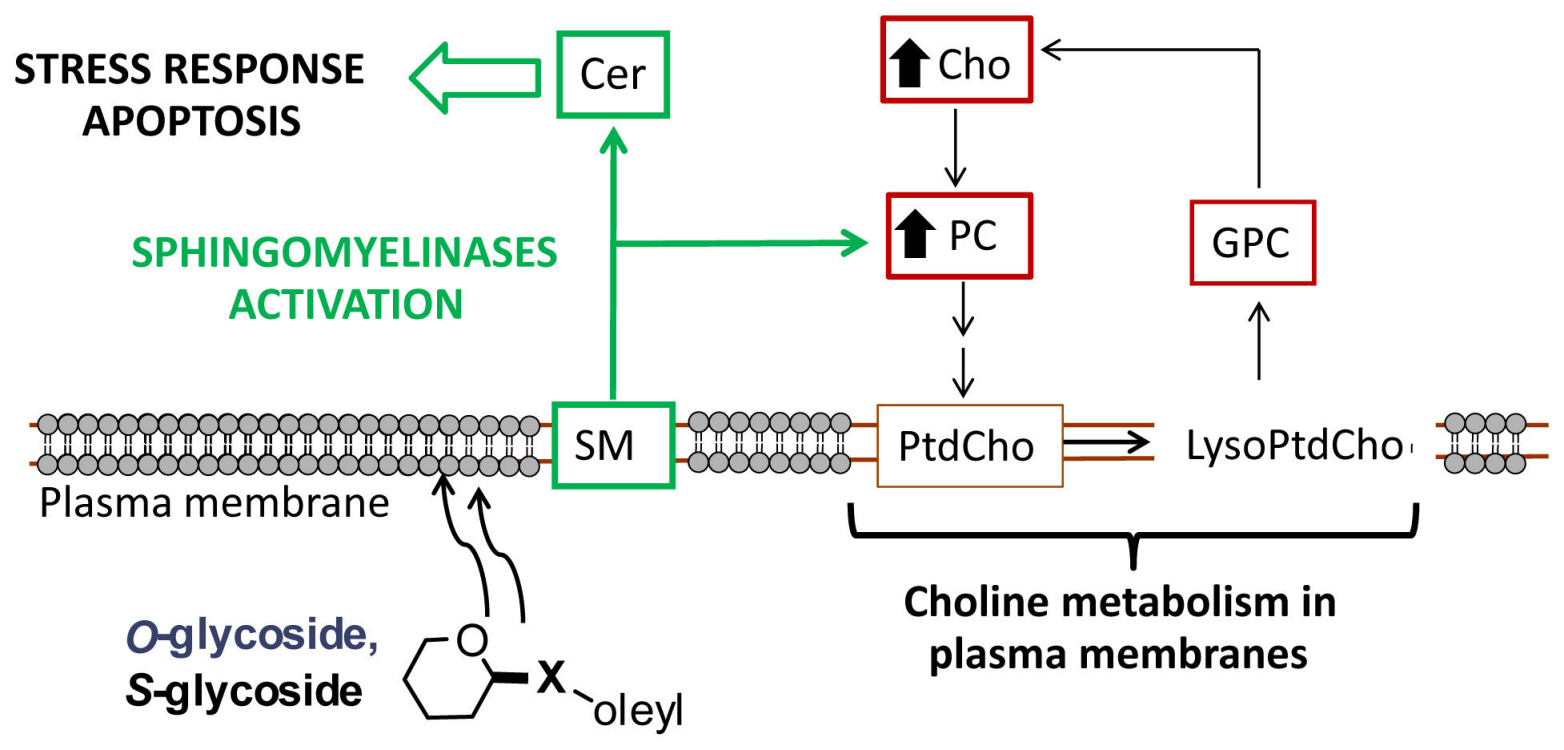
Schematic representation of the possible mechanism for deregulated choline cancer metabolism with drug treatment. Black arrows in Cho and PC indicate a rise in the levels of these metabolites detected in this study.

Abnormal choline metabolism has been associated with oncogenesis and tumor progression. The enzymes involved in choline metabolism have been extensively studied as attractive targets for drug development [25,26]. After malignant transformation, the modulation of enzymes that control anabolic and catabolic pathways causes increased levels of choline-containing precursors and breakdown products of membrane phospholipids. The increased levels in ^t^Cho present in most cancers are associated with proliferation; besides, as emphasized in a recent review [27], there are complex reciprocal interactions between oncogenic signaling and choline metabolism. However, there are considerable differences in the relative contribution of individual choline-containing metabolites between cancer subtypes and, also, after drug treatments. The low PC and high GPC levels observed in non-malignant cells change to high PC and low GPC levels after malignant transformation [28]. Recent studies of highly malignant human breast cancer cell lines and in human tumors displaying malignant choline metabolite profiles (high PC and low GPC levels) have shown increased expression of glycerophosphocholine phosphodiesterase, which catalyzes the degradation of GPC to free choline and glycerol-3-phosphate. The authors suggest that this enzyme is most likely involved in membrane PtdCho metabolism [29]. Our results indicate that C6 glioma cell treatment with glycosides produces an increase in Cho and PC but not a decrease in GPC, which suggests that other choline-related metabolic pathways are also involved, at least in these cells. Sphingomyelinases, which have emerged as important players in modulating oxidative stress-mediated neural cell death in brain tissue [30], are preferentially located in plasma membranes and lysosomes/endosomes [20] and catalyze the cleavage of the phosphodiester bond in SM to form ceramide and PC. Thus, these enzymes could be involved in cancer pathogenesis and may potentially contribute to the elevated PC levels that constitute the cholinic phenotype (Figure 3). Inhibition of choline kinase-α has been reported to increase ceramide levels and, therefore, sphingomyelinases may have a role in the action of choline kinase-α inhibitors as antitumoral drugs [31]. 

Therefore, we suggest that treatment with compounds **1** and **2** could result in activation of sphingomyelinases (Figure 3), that may contribute to the high levels of PC detected here with proton NMR spectroscopy and to the increase in ceramide levels observed previously [8]. This activation of sphingomyelinases could, directly or indirectly, lead to activation of apoptosis mediated by endoplasmic reticulum stress. 

There are other conditions where increased levels of PC in cells have been detected. For instance, a recent study showed that the concentration of PC in glioma cells increased after nutrient deprivation [32]. Alterations in the PC/GPC ratio indicate changes in choline homeostasis [33] and PtdCho metabolism [34,35]. An increase in ^t^Cho, associated with increased GPC levels, was identified in growth arrested human prostate carcinoma [36], suggesting that the relative alteration in choline containing metabolites and, in particular, PC/GPC may be a reliable indicator of cell stress. Changes in individual choline metabolites after different types of treatment have been reported; however, neither a general pattern of responses of the individual choline containing metabolites, nor of ^t^Cho is documented [4]. We have observed that the treatment of C6 glioma cells with compounds **1** and **2** produces an increase in ^t^Cho, mainly due to an increase in levels of Cho and PC, but not GPC. Thus, cancer cell treatments with anti-proliferative compounds **1** and **2**, which result in an increase in ^t^Cho, could be effective due to the activation of sphingomyelinases that induce endoplasmic reticulum stress responses causing apoptosis, but could have the opposite effect of other chemotherapies which reduce ^t^Cho levels.

Other metabolite that significantly increases upon treatment with the two compounds, and does so in a dose-response manner, is acetate. Acetate is a short chain fatty acid, an essential building block for synthesis of many metabolites. It has been detected by NMR in normal rat brain, human resected tissues and cell cultures [11]. The increased levels of acetate could be produced under insufficient oxygen supply by the mediation of cytosolic acetyl acetyl-CoA synthetase [37].

#### NMR analysis of intact tumor tissues

**Figure 4:**
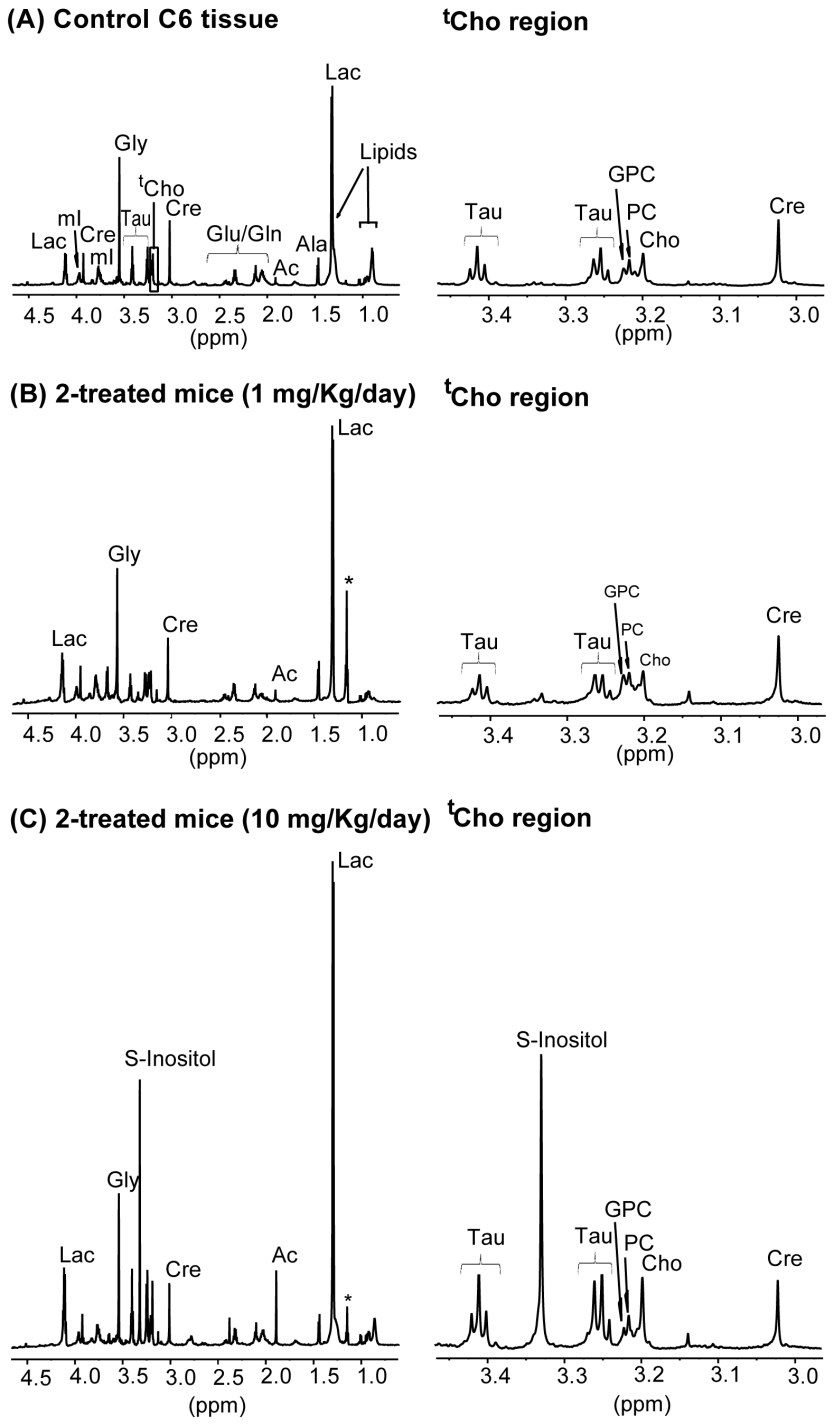
Representative ^1^H HR-MAS NMR spectra of C6 tumor core tissue. (A) Tumor of control mice (B) Tumor of **2**-treated mice (1 mg/Kg) (C) Tumor of **2**-treated mice (10 mg/Kg). The complete aliphatic region is shown on the left panels, and expansion of the taurine and creatine regions on the right, *asterisks*: ethanol contamination.

The activity of compounds **1** and **2** on tumor growth was evaluated using an orthotopic model in female nude mice. Although glycoside **1** presented a higher anti-proliferative activity on C6 cells in culture than compound **2**, only tumors treated with the highest dose of thioglycoside **2** were significantly reduced in size compared to controls [9]. After the *in vivo* experiment, two different tumor biopsy samples were dissected and analyzed: samples from the core and samples from the periphery of the tumor. As expected from its contact with the implantation site, tissue samples from the tumor periphery presented greater amounts of lipids and increased heterogeneity (not shown); therefore, we focused on the analysis of core tumor samples. A representative ^1^H HR-MAS NMR spectrum of control C6 tumor core tissue is shown in Figure 4. 

Several new and relevant resonances appear in the tumor spectra, which are well resolved and can be assigned due to the high resolution of the spectra, such as the singlet a 3.55 ppm, assigned to the two methylene protons of glycine (Gly), an amino acid distributed throughout the brain and nervous system, and characteristic of glial tumors [38]. The two triplets at 3.45 and 3.25 ppm correspond to taurine (Tau) (2-aminoethanesulfonic acid). The singlet centered at 3.33 ppm was ascribed to scyllo-inositol (S-Inositol) [39], which is the second most abundant isomer of inositol found in mammals, and can be detected in human brain using magnetic resonance spectroscopy [40] and in human prostate using magnetic resonance spectroscopic imaging [41]. However, this resonance has been detected only in a few samples (three of six samples) of the tumors treated with the highest dose of thioglycoside **2** (1.5±1.7 intensity of S-Inositol relative to creatine, Table 2). The concentration of S-Inositol seems to be tightly coupled to the myoinositol (mI) concentration at a ratio of 12 mI: 1 S-Inositol [39], so that the elevated S-Inositol might provide indirect evidence of elevated mI. By contrast, our results showed no variations in mI content of tumor tissues with any treatment respect to controls, following its resonance at 3.97 ppm (0.21±0.03 and 0.24±0.04 intensity of mI relative to creatine, for controls and for treatment with 10 mg/Kg/day of compound **2**, respectively). The prominent S-Inositol peak in these tumor tissues deserves further study before its clinical significance can be assessed. Concerning the choline region, a new peak resolved only in tissue spectra and not in cell spectra appeared at 3.21 ppm. This peak has been tentatively assigned to carnitine (Carn), which is a low molecular metabolite containing (as the 3 choline-containing metabolite signals) the group –N(CH_3_)_3_ in its chemical structure. The most apparent difference of tissue spectra (Figure 4) when compared to the corresponding cells in culture (Figure 2) is the detection of significantly smaller levels of total choline (a maximum of 2 in tissue, versus 7 in cells, when normalized to creatine methyl intensities).

**Table 2:** Quantification of relevant metabolites and their variation^(a)^ upon tumor treatment with compounds 1-2 at the indicated doses administrated per day.

**Metabolite**	**Signal**	**Control**	**1** (10 mg/Kg)	**2** (1 mg/Kg)	**2** (10 mg/Kg)
Gly	3.55, s	2.17±0.15	2.12±0.32	2.23±0.17	2.00±0.33
Tau	3.42, t	0.43±0.11	0.48±0.06	0.40±0.07	0.74±0.29
S-Inositol	3.33, s	-	0.8±1.70	0.12±0.07	1.50±1.70
GPC	3.23, s	0.29±0.05	0.36±0.04	0.38±0.04	0.32±0.10
PC	3.22, s	0.39±0.09	0.43±0.05	0.46±0.05*	0.54±0.05*
Cho	3.20, s	0.49±0.09	0.55±0.10	0.47±0.07	0.83±0.20*
^t^Cho	3.2, m	1.17±0.23	1.60±0.19	1.51±0.16	1.96±0.35
PC/GPC		1.34±0.50	1.19±0.27	1.21±0.26	1.69±0.68
Carn	3.21, s	0.23±0.02	0.26±0.03	0.26±0.03	0.27±0.03
Lac	4.11, q	0.51±0.05	0.70±0.07**	0.63±0.11	0.97±0.34
Ac	1.91, s	0.1±0.02	0.16±0.01	0.20±0.16	0.53±0.40

^a^ Relative intensity to creatine (I/I_creatine_). Results represent the mean ± SD of six experiments. Statistically significant differences of each treatment respect to control values are indicated as follows: * p ≤ 0.05; ** p ≤ 0.01).

Significant variations in the spectra of tumors were detected only in tumors treated with the highest dose of **2** (10 mg/Kg/day), whereas treatment with **1**, which is susceptible of enzymatic hydrolysis *in vivo*, resulted in low, non-significant and variable changes. The most significant metabolic change detected in treated tumor tissues with the highest dose of **2** was a significant increase in Cho (2 times) and PC (1.4 times) levels, similar to the results obtained in cell culture; the ratio PC/GPC was also higher (1.69±0.68 versus 1.34±0.5) in treated tumors (Table 2). On the other hand, the tumors treated with glycoside **1**, which had *in vitro* activity on cells but was inactive *in vivo* (as judged by its inability to reduce tumor volume, probably due, among other factors, to its hydrolysis *in vivo*) [9], showed NMR spectra consistently similar to those of control tissue samples. These results support the hypothesis that the increases in ^t^Cho detected by NMR spectroscopy are associated to the antitumor activity of the compounds. 

Taurine levels increased almost two-fold in tumors treated with the highest dose of thioglycoside with respect to control values, although the difference was not statistically significant (0.43±0.11 to 0.74±0.29, Table 2). Taurine concentration has been correlated with apoptosis in astrocytomas and has been suggested as an indicator for the clinical monitoring of glioma apoptosis [42], supporting the hypothesis that the activity of the thioglycoside could be associated to its ability to induce tumor apoptosis.

**Figure 5:**
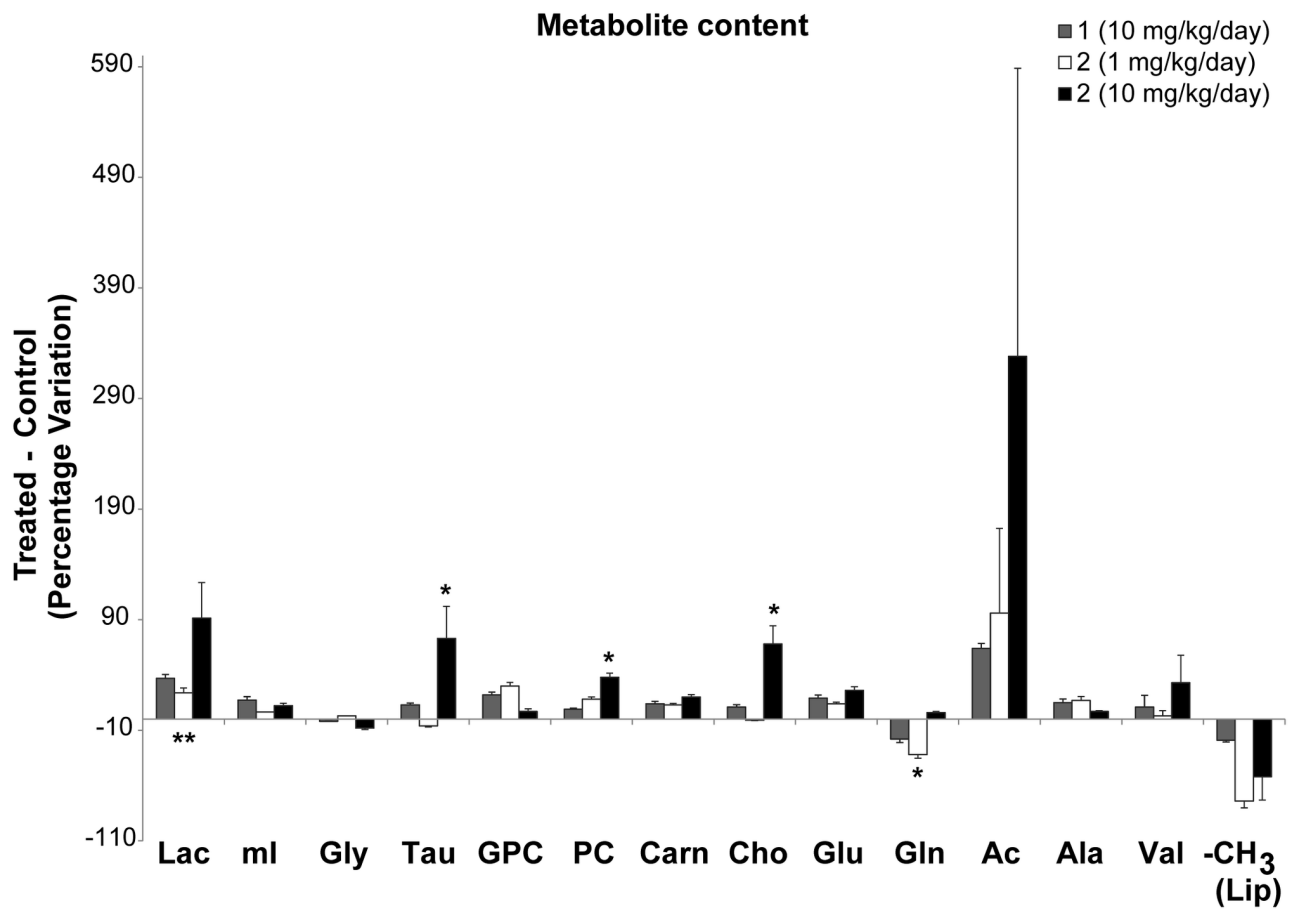
Histograms summarizing the percentage differences detected in several metabolites between C6 tumor core tissue treated with glycoside 1 (10 mg/Kg/day) and 2 (1 or 10 mg/Kg/day) and the non-treated control. Each value in the histogram represents the mean ± SD of six separate experiments. Positive values indicate the increased presence of a metabolite in tumors with respect to controls. Asterisks indicate statistically significant differences in a particular metabolite evaluated at p < 0.05. *, p ≤ 0.05; **, p ≤ 0.01.

As in the case of cell cultures, treatment with the lowest dose of compound **2** caused no significant differences in the levels of Glu, Ala and Val, whereas, Gln levels decreased by 32 % as compared to non-treated tumors (Figure 5). Gln is a precursor and storage form of Glu, which slightly increased with treatments. These changes could be associated with increased amino acid influx into de TCA cycle (for example of Glu in the form of α-ketoglutarate), providing an alternative (non glycolytic) energy source to cancer cells [43], which might be under stress and/or hypoxia after compound-treatment, and previous to cell death.

The tumors treated with compounds presented additional variations in lactate and acetate signals compared to controls (Figure 5). Although these variations were not statistically significant, they indicate a trend that deserves discussion. Acetate concentration appeared to present a dose-response dependence with thioglycoside **2**-treatment. Alterations in acetate levels have been reported in tumoral tissues, although the variations depended on the type of tumor. Thus, acetate levels seemed reduced in lung tumors compared to control tissue [44], whereas they appeared increased in biopsies of child neuroblastoma [45]. Lactate also tended to increase after treatment, particularly with compound **2**. Lactate is the end product of anaerobic glycolysis, normally present in brain tissue at low concentrations [46]; however, a concentration increase rapidly follows hypoxia. Both metabolites have been related to the anaerobic energy metabolism of cancer cells [47,48]. In this respect, the increased levels of acetate and lactate in the tumors treated with compound **2** raise the possibility that **2** has an antiangiogenic effect, leading to a more hypoxic tumor microenvironment. The promotion of anaerobic metabolism by thioglycoside **2** is more pronounced in the core of the tumor than its periphery, as confirmed by the NMR spectra of the tissue from this region, which showed smaller increases in acetate and lactate than in the core of the tumor (Table 3). Along these lines, different studies have reported that hypoxia can promote apoptosis in tumor cells [49] and plays a crucial role in regulating the altered lipid metabolism in human glioblastoma cells [50,51]. 

**Table 3:** Quantification of acetate and lactate and their variation^(a)^ upon tumor treatment with compounds 1-2 at the indicated doses administrated per day on tissues from the periphery of tumor biopsies.

**Metabolite**	**Signal**	**Control**	**1** (10 mg/Kg)	**2** (1 mg/Kg)	**2** (10 mg/Kg)
Lac	4.11, q	0.57±0.25	0.59±0.11	0.62±0.12	0.64±0.19
Ac	1.91, s	0.12±0.05	0.15±0.06	0.15±0.07	0.19±0.08

a Relative intensity to creatine (I/I_creatine_). Results represent the mean ± SD of six experiments

## Conclusion

Based on the analysis of intact C6 cells and tumor tissues using ^1^H HR-MAS NMR spectroscopy, we have established a good correlation between the changes in metabolic profiles for *in vitro* anti-proliferative activity and *in vivo* antitumor activity brought about by tumor treatment with glycoside derivatives. Specifically, the activity of antitumoral compounds was associated with an increase in choline and phosphocholine, observed in both cell culture and tumor tissues. Other metabolites also change upon tumor treatment in mice, such as taurine, considered a biomarker of apoptosis. Acetate and lactate, metabolites that have been related to the anaerobic energy metabolism of cancer cells, also increase with increasing antitumoral dose. Thus, these results indicate that the mode of action of the glycoside involves, at least partially, alteration of phospholipid metabolism, and results in cell death. This study is another illustration of the potential of proton NMR spectroscopy-based metabolomics to help uncover metabolic processes, as well as to compare *in vitro* and *in vivo* models of human response to pharmacological treatment.

## References

[1] García-Álvarez I, Fernández-Mayoralas A, Garrido L (2011) Effect of Drugs in Cells and Tissues by NMR spectroscopy. Curr Top Med Chem 11: 27–42. doi:10.2174/156802611793611841. PubMed: 20809892. 20809892

[2] Lindon JC, Beckonert OP, Holmes E, Nicholson JC (2009) High resolution magic angle spinning NMR spectroscopy: Application to biomedical studies. Prog NMR Spectrosc 55: 79–100. doi:10.1016/j.pnmrs.2008.11.004.

[3] Sitter B, Bathen T, Tessem M, Gribbestad I (2009) High resolution magic angle spinning (HR MAS) MR spectroscopy in metabolic characterization of human cancer. Prog NMR Spectrosc 54: 239–254. doi:10.1016/j.pnmrs.2008.10.001.

[4] Moestue S, Sitter B, Bathen TF, Tessem MB, Gribbestad IS (2011) HR MAS MR spectroscopy in Metabolic Characterization of Cancer. Curr Top Med Chem 11: 2–26. doi:10.2174/156802611793611869. PubMed: 20809888. 20809888

[5] García-Álvarez I, Corrales G, Doncel-Pérez E, Nieto-Sampedro M, Fernández-Mayoralas A (2007) Design and Synthesis of Glycoside Inhibitors of Glioma and Melanoma Growth. J Med Chem 50: 364–373. doi:10.1021/jm0611556. PubMed: 17228879. 17228879

[6] Bello C, Dal Bello G, Cea M, Nahimana A, Aubry D et al. (2011) Anti-cancer activity of 5-*O*-alkyl 1,4-imino-1,4-dideoxyribitols. Bioorg Med Chem 19: 7720-7727. doi:10.1016/j.bmc.2011.07.053. PubMed: 22079865. 22079865

[7] García-Álvarez I, Garrido L, Doncel-Pérez E, Nieto-Sampedro M, Fernández-Mayoralas A (2009) Detection of Metabolite Changes in C6 Glioma Cells Cultured with Antimitotic Oleyl Glycoside by 1 H MAS NMR. J Med Chem 52: 1263–1267. doi:10.1021/jm8012807. PubMed: 19199478. 19199478

[8] García-Álvarez I, Egido-Gabás M, Romero-Ramírez L, Doncel-Pérez E, Nieto-Sampedro M et al. (2011) Lipid and ganglioside alterations in tumor cells treated with antimitotic oleyl glycoside. Mol Biosyst 7: 129–138. doi:10.1039/c0mb00125b. PubMed: 21057675. 21057675

[9] García-Álvarez I, Groult H, Casas J, Barreda-Manso MA, Yanguas-Casás N et al. (2011) Synthesis of Antimitotic Thioglycosides: in Vitro and in Vivo Evaluation of their Anticancer Activity. J Med Chem 54: 6949-6955. doi:10.1021/jm200961q. PubMed: 21866909. 21866909

[10] Rocha CM, Barros AS, Gil AM, Goodfellow BJ, Humpfer E et al. (2010) Metabolic Profiling of Human Lung Cancer Tissue by 1 H High Resolution Magic Angle Spinning (HRMAS) NMR Spectroscopy. J Proteome Res 9: 319-332. doi:10.1021/pr9006574. PubMed: 19908917. 19908917

[11] Govindaraju V, Young K, Maudsley AA (2000) Proton NMR chemical shifts and coupling constants for brain metabolites. NMR Biomed 13: 129–153. doi:10.1002/1099-1492(200005)13:3. PubMed: 10861994. 10861994

[12] Moore DS, McCabe GP (1989) Introduction to the Practice of Statistics. New York: W.H. Freeman & Co.

[13] Brown BW Jr, Hollander M (1977) Statistics: A Biomedical Introduction. New York: John Wiley & Sons, Inc.

[14] Mendenhall W (1989) Introduction to Probability and Statistics. 7th edn. Boston, Massachusetts: Duxbury Press.

[15] Quintero M, Cabañas ME, Arús C (2007) A posible cellular explanation for the NMR-visible mobile lipid (ML) changes in cultured C6 glioma cells with growth. Biochim Biophys Acta 1771: 31–44. doi:10.1016/j.bbalip.2006.10.003. PubMed: 17150408. 17150408

[16] Griffin JL, Bollard M, Nicholson JK, Bhakoo K (2002) Spectral profiles of cultured neuronal and glial cells derived from HRMAS 1H NMR spectroscopy. NMR Biomed 15: 375-384. doi:10.1002/nbm.792. PubMed: 12357551. 12357551

[17] Griffin JL, Williams HJ, Sang E, Nicholson JK (2001) Abnormal lipid profile of dystrophic cardiac tissue as demonstrated by one and two dimensional magic angle spinning 1H NMR spectroscopy. Magn Reson Med 46: 249–255. doi:10.1002/mrm.1185. PubMed: 11477627. 11477627

[18] Gallinger A, Biet T, Pellerin L, Peters T (2011) Insights into Neuronal Cell Metabolism Using NMR Spectroscopy: Uridyl Diphosphate *N*-Acetyl-Glucosamine as a Unique Metabolic Marker. Angew Chem Int Ed 50: 11672–11674 doi:10.1002/anie.201104836. PubMed: 22012807. 22012807

[19] Morales A, Lee H, Goñi FM, Kolesnick R, Fernandez-Checa JC (2007) Sphingolipids and cell death. Apoptosis 12: 923-939. doi:10.1007/s10495-007-0721-0. PubMed: 17294080. 17294080

[20] Ohanian J, Ohanian V (2001) Sphingolipids in mammalian cell signalling. Cell Mol Life Sci 58: 2053–2068. doi:10.1007/PL00000836. PubMed: 11814056. 11814056 PMC11337328

[21] Gómez-Muñoz A, Kong JY, Salh B, Steinbrecher UP (2004) Ceramide-1-phosphate blocks apoptosis through inhibition of acid sphingomyelinase in macrophages. J Lipid Res 45: 99-105. PubMed: 14523050. 14523050 10.1194/jlr.M300158-JLR200

[22] Hannun YA, Obeid LM (2002) The Ceramide-centric universe of lipid-mediated cell regulation: stress encounters of the lipid kind. J Biol Chem 277: 25847–25850. doi:10.1074/jbc.R200008200. PubMed: 12011103. 12011103

[23] Andrieu-Abadie N, Gouazé V, Salvayre R, Levade T (2001) Ceramide in apoptosis signaling: relationship with oxidative stress. Free Radic Biol Med 31: 717–728. doi:10.1016/S0891-5849(01)00655-4. PubMed: 11557309. 11557309

[24] Brindle KM (2002) Detection of apoptosis in tumors using magnetic resonance imaging and spectroscopy. Adv Enzyme Regul 42: 101-112. doi:10.1016/S0065-2571(01)00025-5. PubMed: 12123709. 12123709

[25] Gallego-Ortega D, Gómez del Pulgar T, Valdés-Mora F, Cebrián A, Lacal JC (2011) Involvement of human choline kinase alpha and beta in carcinogenesis: a different role in lipid metabolism and biological functions. Adv Enzyme Regul 51: 183-194. doi:10.1016/j.advenzreg.2010.09.010. PubMed: 21035492. 21035492

[26] Rodríguez-González A, Ramírez de Molina A, Benítez-Rajal J, Lacal JC (2003) Phospholipase D and choline kinase: their role in cancer development and their potential as drug targets. Prog Cell Cycle Res 5: 191-201. PubMed: 14593713. 14593713

[27] Glunde K, Bhujwalla ZM, Ronen SM (2011) Choline metabolism in malignant transformation. Nat Rev Cancer 11: 835-848. PubMed: 22089420. 22089420 10.1038/nrc3162 PMC4337883

[28] Aboagye EO, Bhujwalla ZM (1999) Malignant transformation alters membrane choline phospholipid metabolism of human mammary epithelial cells. Cancer Res 59: 80-84. PubMed: 9892190. 9892190

[29] Cao MD, Döpkens M, Krishnamachary B, Vesuna F, Gadiya MM et al. (2012) Glycerophosphodiester phosphodiesterase domain containing 5 (GDPD5) expression correlates with malignant choline phospholipid metabolite profiles in human breast cancer. NMR Biomed 25: 1033–1042. doi:10.1002/nbm.2766. PubMed: 22279038. 22279038 PMC4126590

[30] Farooqui AA, Horrocks LA, Farooqui T (2007) Interactions Between Neural Membrane Glycerophospholipid and Sphingolipid Mediators: A Recipe for Neural Cell Survival or Suicide. J Neurosci Res 85: 1834–1850. doi:10.1002/jnr.21268. PubMed: 17393491. 17393491

[31] Rodríguez-González A, Ramirez de Molina A, Fernández F, Lacal JC (2004) Choline kinase inhibition induces the increase in ceramides resulting in a highly specific and selective cytotoxic antitumoral strategy as a potential mechanism of action. Oncogene 23: 8247–8259. doi:10.1038/sj.onc.1208045. PubMed: 15378008. 15378008

[32] Mirbahai L, Wilson M, Shaw CS, McConville C, Malcomson RDG et al. (2011) 1H magnetic resonance spectroscopy metabolites as biomarkers for cell cycle arrest and cell death in rat glioma cells. Int J Biochem Cell B 43: 990–1001. doi:10.1016/j.biocel.2010.07.002. 20633697

[33] Lutz NW (2005) From metabolic to metabolomic NMR spectroscopy of apoptotic cells. Metabolomics 1: 251–268. doi:10.1007/s11306-005-0005-z.

[34] Podo F (1999) Tumour phospholipid metabolism. NMR Biomed 12: 413–439. doi:10.1002/(SICI)1099-1492(199911)12:7. PubMed: 10654290. 10654290

[35] Morse DL, Raghunand N, Sadarangani P, Murthi S, Job C et al. (2007) Response of choline metabolites to docetaxel therapy is quantified in vivo by localized 31P MRS of human breast cancer xenografts and in vitro by high-resolution 31P NMR spectroscopy of cell extracts. Magn Reson Med 58: 270–280. doi:10.1002/mrm.21333. PubMed: 17654590. 17654590

[36] Milkevitch M, Shim H, Pilatus U, Pickup S, Wehrle JP et al. (2005) Increases in NMR-visible lipid and glycerophosphocholine during phenylbutyrate induced apoptosis in human prostate cancer cells. Biochim Biophys Acta 1734: 1–12. doi:10.1016/j.bbalip.2005.01.008. PubMed: 15866478. 15866478

[37] Yoshii Y, Furukawa T, Yoshii H, Mori T, Kiyono Y et al. (2009) Cytosolic acetyl-CoA synthetase affected tumor cell survival under hypoxia: the possible function in tumor acetyl-CoA/acetate metabolism. Cancer Sci 100: 821-827. doi:10.1111/j.1349-7006.2009.01099.x. PubMed: 19445015. 19445015 PMC11158093

[38] Kinoshita Y, Yokota A (1997) Absolute Concentrations of Metabolites in Human Brain Tumors Using In Vitro Proton Magnetic Resonance Spectroscopy. NMR Biomed 10: 2–12. doi:10.1002/(SICI)1099-1492(199701)10:1. PubMed: 9251109. 9251109

[39] Michaelis T, Helms G, Merboldt KD, Hänicke W, Bruhn H et al. (1993) Identification of Scyllo-inositol in proton NMR spectra of human brain in vivo. NMR Biomed 6: 105-109. doi:10.1002/nbm.1940060116. PubMed: 8384468. 8384468

[40] Kaiser LG, Schuff N, Cashdollar N, Weiner MW (2005) Scyllo-inositol in normal aging human brain: 1 H magnetic resonance spectroscopy study at 4 Tesla. NMR Biomed 18: 51–55. doi:10.1002/nbm.927. PubMed: 15468140. 15468140 PMC1820854

[41] Venugopal N, McCurdy B, Al Mehairi S, Alamri A, Sandhu GS et al. (2012) Short echo time in vivo prostate 1H-MRSI. Magn Reson Imaging 30: 195–204. doi:10.1016/j.mri.2011.09.020. PubMed: 22154684. 22154684

[42] Opstad KS, Bell BA, Griffiths JR, Howe FA (2009) Taurine: a potential marker of apoptosis in gliomas. Br J Cancer 100: 789–794. doi:10.1038/sj.bjc.6604933. PubMed: 19223899. 19223899 PMC2653765

[43] Somashekar BS, Kamarajan P, Danciu T, Kapila YL, Chinnaiyan AM et al. (2011) Magic Angle Spinning NMR-Based Metabolic Profiling of Head and Neck Squamous Cell Carcinoma Tissues. J Proteome Res 10: 5232-5241. doi:10.1021/pr200800w. PubMed: 21961579. 21961579 PMC3208743

[44] Duarte IF, Rocha CM, Barros AS, Gil AM, Goodfellow BJ et al. (2010) Can nuclear magnetic resonance (NMR) spectroscopy reveal different metabolic signatures for lung tumours? Virchows Arch 457: 715–725. doi:10.1007/s00428-010-0993-6. PubMed: 20941505. 20941505

[45] Imperiale A, Elbayed K, Moussallieh FM, Neuville A, Piotto M et al. (2011) Metabolomic Pattern of Childhood Neuroblastoma Obtained by 1 H-High-Resolution Magic Angle Spinning (HRMAS) NMR Spectroscopy. Pediatr Blood Cancer 56: 24–34. doi:10.1002/pbc.22668. PubMed: 20949594. 20949594

[46] Veech RL (1991) The metabolism of lactate. NMR Biomed 4: 53–58. doi:10.1002/nbm.1940040204. PubMed: 1859786. 1859786

[47] Mazzio EA, Smith B, Soliman KFA (2010) Evaluation of endogenous acidic metabolic products associated with carbohydrate metabolism in tumor cells. Cell Biol Toxicol 26: 177–188. doi:10.1007/s10565-009-9138-6. PubMed: 19784859. 19784859 PMC2886276

[48] Wilson WR, Hay MP (2011) Targeting hypoxia in cancer therapy. Nat Rev Cancer 11: 393-410. doi:10.1038/nrc3064. PubMed: 21606941. 21606941

[49] Kunz M, Ibrahim SM (2003) Molecular responses to hypoxia in tumor cells. Mol Cancer 2: 1-13. doi:10.1186/1476-4598-2-1. PubMed: 12537587. 12740039 PMC155638

[50] Laurenti G, Benedetti E, D’Angelo B, Cristiano L, Cinque B et al. (2011) Hypoxia Induces Peroxisome Proliferator-Activated Receptor a (PPARa) and Lipid Metabolism Peroxisomal Enzymes in Human Glioblastoma Cells. J Cell Biochem 112: 3891–3901. doi:10.1002/jcb.23323. PubMed: 21866563. 21866563

[51] Benedetti E, Galzio R, Laurenti G, D'Angelo B, Melchiorre E et al. (2010) Lipid metabolism impairment in human gliomas: expression of peroxisomal proteins in human gliomas at different grades of malignancy. Int J Immunopathol Pharmacol 23: 235-246. PubMed: 20378009. 20378009 10.1177/039463201002300121

